# Deciphering the genetic network and programmed regulation of antimicrobial resistance in bacterial pathogens

**DOI:** 10.3389/fcimb.2022.952491

**Published:** 2022-11-23

**Authors:** Thandavarayan Ramamurthy, Amit Ghosh, Goutam Chowdhury, Asish K. Mukhopadhyay, Shanta Dutta, Shin-inchi Miyoshi

**Affiliations:** ^1^ Division of Bacteriology, ICMR-National Institute of Cholera and Enteric Diseases, Kolkata, India; ^2^ Collaborative Research Centre of Okayama University for Infectious Diseases at ICMR- National Institute of Cholera and Enteric Diseases, Kolkata, India; ^3^ Graduate School of Medicine, Dentistry and Pharmaceutical Sciences, Okayama University, Okayama, Japan

**Keywords:** CRiSPR/Cas, efflux pump, mobile genetic elements, phages, quorum sensing, toxin-antitoxin, Two- component system, antimicrobial resistance

## Abstract

Antimicrobial resistance (AMR) in bacteria is an important global health problem affecting humans, animals, and the environment. AMR is considered as one of the major components in the “global one health”. Misuse/overuse of antibiotics in any one of the segments can impact the integrity of the others. In the presence of antibiotic selective pressure, bacteria tend to develop several defense mechanisms, which include structural changes of the bacterial outer membrane, enzymatic processes, gene upregulation, mutations, adaptive resistance, and biofilm formation. Several components of mobile genetic elements (MGEs) play an important role in the dissemination of AMR. Each one of these components has a specific function that lasts long, irrespective of any antibiotic pressure. Integrative and conjugative elements (ICEs), insertion sequence elements (ISs), and transposons carry the antimicrobial resistance genes (ARGs) on different genetic backbones. Successful transfer of ARGs depends on the class of plasmids, regulons, ISs proximity, and type of recombination systems. Additionally, phage-bacterial networks play a major role in the transmission of ARGs, especially in bacteria from the environment and foods of animal origin. Several other functional attributes of bacteria also get successfully modified to acquire ARGs. These include efflux pumps, toxin-antitoxin systems, regulatory small RNAs, guanosine pentaphosphate signaling, quorum sensing, two-component system, and clustered regularly interspaced short palindromic repeats (CRISPR) systems. The metabolic and virulence state of bacteria is also associated with a range of genetic and phenotypic resistance mechanisms. In spite of the availability of a considerable information on AMR, the network associations between selection pressures and several of the components mentioned above are poorly understood. Understanding how a pathogen resists and regulates the ARGs in response to antimicrobials can help in controlling the development of resistance. Here, we provide an overview of the importance of genetic network and regulation of AMR in bacterial pathogens.

## Introduction

Antimicrobial resistance (AMR) is a major public health concern, which has been continued to increase primarily due to inappropriate use of antibiotics in human health and in the production of food animals. An estimated 4·95 million deaths were associated with AMR in 2019, which included 1·27 million deaths directly attributable to pathogenic bacteria (ARC, 2022). Predictions of the rising magnitude of AMR-associated death might be around 10 million people by 2050, if no interventions are applied (Balouiri et al., 2016).

Since their discovery, antimicrobials have been successfully used in the treatment of different infectious diseases. However, the efficacy of many antimicrobials is progressively being compromised with the increase of resistance in disease-associated bacteria. The rapid emergence and spread of AMR continue to be a challenging problem, especially in clinical settings, animal farming, and food manufacturing (Queenan et al., 2016; Matthiessen et al., 2022). The emergence of multidrug-resistant (MDR), extensively drug-resistant and pan-drug-resistant bacterial strains severely limits existing therapeutic options (Magiorakos et al., 2012; Poulakou et al., 2014). Besides the development of new drugs, strategies to prevent the development and spread of resistance are being extensively explored (Hashempour-Baltork et al., 2019). These approaches require a clear understanding the mechanisms of AMR and the role of environmental factors that contribute to the development of resistance.

**Figure 1:**
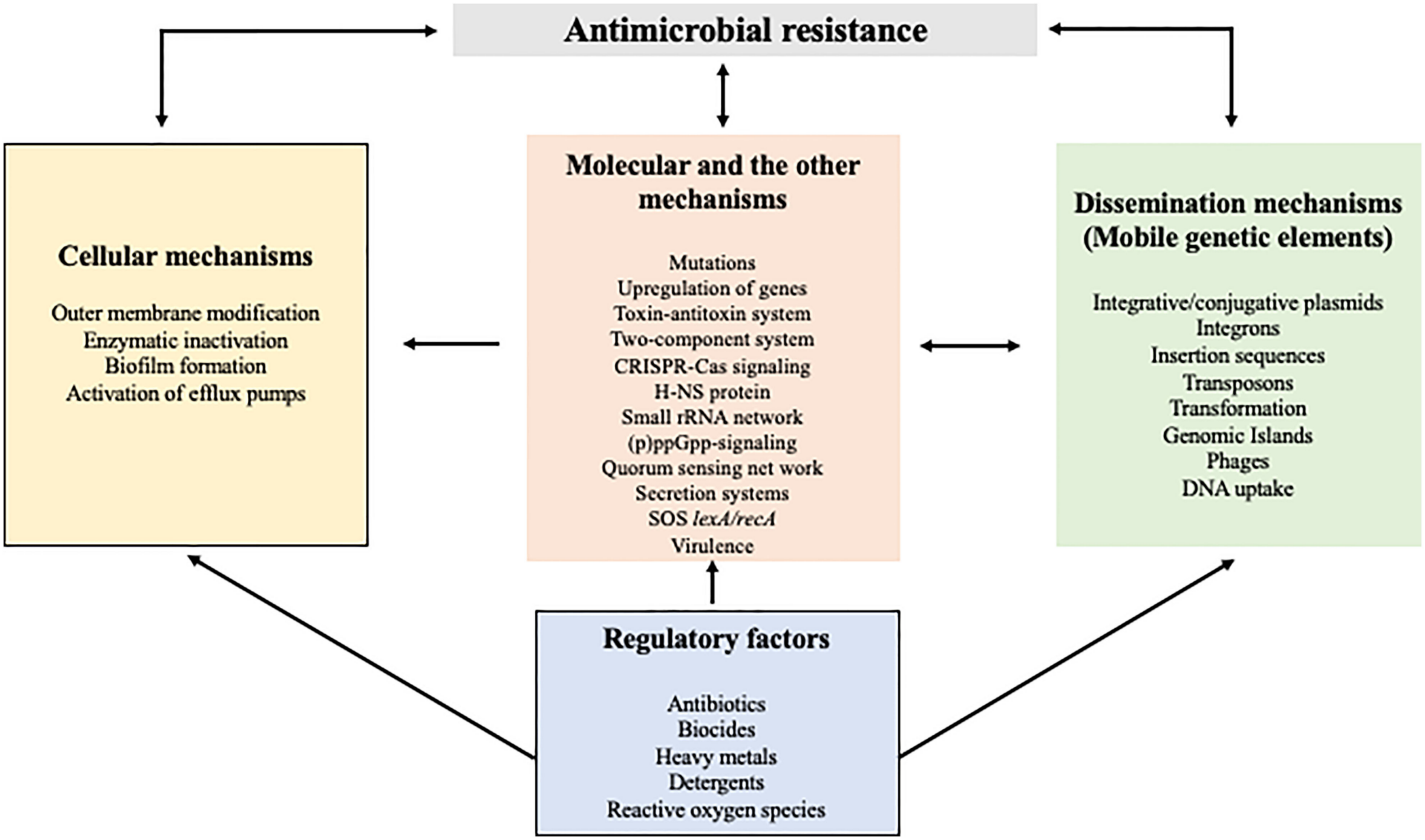
Common mechanisms and their interaction in the dissemination of antimicrobial resistance. As shown in different boxes, cellular and molecular mechanisms as well as mobile genetic elements are responsible for the acquisition and dissemination of AMR. The regulatory factors that prevail in the environment directly or indirectly influence many of the AMR mechanism.

The founder effect, fitness costs within the host, and their ecological association influence the success of AMR transmission. In the presence of antibiotic selective pressure, bacteria develop several levels of defense, which include structural changes of the bacterial outer membrane, enzymatic mechanisms for antibiotic-inactivation, gene upregulation, mutations, adaptive resistance, and formation of resistant phenotypes and biofilm (Abushaheen et al., 2020). Several such factors, and their interactions between them are shown in **Figure 1**. Resistance might occur either through a single mechanism against multiple antibiotics or multiple mechanisms against a single antibiotic.

Knowledge on how a pathogen becomes resistant to antimicrobials by regulating the genes is an important step towards improving the strategies to tackle the AMR problem. Antimicrobial resistance genes (ARGs) may form part of mobile genetic elements (MGEs), which can make the intracellular transfer onto plasmids or gene cassettes (Baquero et al., 2019). Gene regulatory networks permit the bacteria to generate a coordinated response to environmental challenges and shape the epidemiology of AMR. However, the relationship between selection pressure and the evolutionary change of these networks is poorly understood.

Since bacterial virulence genes can act in tandem with AMR and MGEs, there is a need to investigate these gene reservoirs and their mode of transmission. In many findings, it was reported that the co-selection of AMR takes place frequently in the presence of heavy metals, environmental toxicants, and other inorganic agents (Wales and Davies, 2015; Biswas et al., 2021). Non-antibiotic agents such as detergents and heavy metals can induce cross-protection against antimicrobials through the efflux and other systems (Hegstad et al., 2010; Sistrunk et al., 2016; Vats et al., 2022). This review focuses on several important aspects of the genetic networks and regulation of AMR in bacterial pathogens with some known examples.

## Mobile genetic elements

MGEs/transposable elements are the genes that can move within a genome or be transferred from one bacterial species to another. The gene gain or loss due to MGEs could contribute to the adaptation to different environments that eventually help to form a divergent bacterial population (Jørgensen et al., 2015). The expansion of AMR in bacteria is due to intrinsic or acquired mechanisms during unstructured *de novo* mutations and horizontal gene transfer (HGT). Conjugation, transformation, and transduction are the three canonical mechanisms of HGT (García-Aljaro et al., 2017). MGEs can carry ARGs for different categories of antibiotics and play a significant role in their spread within and between bacterial species (Forster et al., 2022). MGEs successfully transfer ARGs *en bloc* on the bacterial chromosome and/or on the broad-host-range plasmids. The linkage of a well-organized gene capture and expression systems, together with the ability for vertical and horizontal transmission of ARGs signifies a powerful defense machinery used by the bacteria to resist several antimicrobials (Bencko and Šíma, 2018; Li et al., 2021). Acquisition of MGEs may have association with fitness cost and maintained due to antimicrobial selective pressure. The presence of MGE is generally alleviated by compensatory mutations in the host chromosome during MGE-host synchronization and coevolution. In the absence of antibiotic pressure, the bacteria can be outcompeted by others that do not carry any MGEs (Depardieu et al., 2007).

Gene clusters gained by HGT in bacterial genomes are referred to as genomic islands (GIs). They generally carry essential genes for genome evolution and environmental adaptation that includes bacterial fitness, metabolism, pathogenesis, AMR, etc (Juhas et al., 2009). Based on their structure and functions, GIs are also considered a superfamily of MGE. GIs have specific features that differentiate them from the core genome, which include the presence of mobility-related genes flanked by direct repeats and specific integration sites (e.g., tDNA [tRNA/tmRNA] gene) (Juhas et al., 2009). For e.g., the *Salmonella* Genomic Island-1 (SGI-1), an MGE found in many enterobacterial isolates, carries several MDR genes encoding resistance to ampicillin, chloramphenicol, streptomycin, sulfonamides, and tetracycline (Amar et al., 2008). However, the insertion position of SGI-1 might differ among many bacterial groups (Siebor and Neuwirth, 2022). The macrolide resistance gene *ermB* was found to be associated with several multidrug resistance genomic islands (MDR-GIs) in *Campylobacter*s (Liu et al., 2019a). In many bacterial species, tetracycline encoding gene *tet*(O) acts as a key integration site for the horizontal transfer of ARGs. During transformation, circular intermediates are formed owing to the presence of two *tet*(O) direct repeats in the terminal parts of MDR-GIs (Friis et al., 2007). Integrons, insertion sequences, transposons, plasmids, bacteriophages are some of the important components that carry several MGEs whose functions including AMR are described below.

### Integrons

Integrons are proficient gene capture and expression system with several gene cassettes. They play an important role in the dissemination of AMR, mainly among Gram-negative bacteria (Cambray et al., 2010). Integrative and conjugative elements (ICEs, also known as conjugative transposons) are self-transmissible MGEs, which have the combined features of prophages as well as transposons for integration and excision from the chromosomes of unrelated bacterial taxa (Akrami et al., 2019). An ICE generally contains a group of cargo genes that encodes metabolic adaptation, virulence, and resistance to antibiotics and/or heavy metals (Johnson and Grossman, 2015). Unlike plasmids, ICEs are not affected by segregational loss and are stably maintained in the host genome. Integration can occur at a single attachment site; often a tRNA gene, or in many locations that are shared by the same class of ICEs to avoid competition for the limited integration sites between different co-infecting ICEs (Cambray et al., 2010). The processing of ICE-DNA for conjugative transfer is similar to that of conjugative plasmids and rolling-circle replication (Thomas et al., 2013). This component includes a type IV secretion system, which makes an intimate contact between the donor and recipient for propagation.

Many anthropogenic factors induce bacterial gene arrangements and mutations, thereby contributing to the dissemination of genes encoding resistance for detergents, heavy metals and antimicrobials (Ghaly et al., 2017). It is well known that class 1 integrons (Intl1) are responsible for the global spread of AMR. The *qacE/qacE*Δ1 (encoding quaternary ammonium compound-resistance) gene cassette that confers resistance to biocides, and the mercury resistance operon (*mer*) has been transmitted by Tn*21* provide a selective benefit in several pathogens (Cambray et al., 2010; Akrami et al., 2019). This resistance mechanism also increases bacterial membrane permeability and stimulates the production of reactive oxygen species (ROS), which possibly helps the transfer of plasmids between bacterial species (Han et al., 2019).

An integron-borne garosamine-specific aminoglycoside resistance encoding *gar* gene has been identified in *Pseudomonas aeruginosa*, *Luteimonas* sp., and *Salmonella enterica* (Böhm et al., 2020). Integron’s specificity to garosamine-containing aminoglycosides may decrease the efficacy of the semi-synthetic aminoglycoside plazomicin and evade the aminoglycoside resistance mechanisms. The gene *gar* is located within integron and adjacent to *aph*(*3′*)*-XV*, *bla*_OXA-2_, and *bla*_VIM-1_ gene cassettes provide resistance to many critically important antibiotics (Böhm et al., 2020). There are many excellent reviews on ICEs covering the structure and functions with reference to the transmission of AMR genes (Johnson and Grossman, 2015; Burrus, 2017; Delavat et al., 2017; Botelho and Schulenburg, 2021).

### Insertion sequence elements

An insertion sequence encodes a transposase enzyme that catalyzes the transposition. Generally, the level of transposase expression influences the frequency of transposition. Insertion sequence elements (ISs) characteristically have concise sequences containing terminal inverted repeats at the boundaries and an open reading frame (ORF) that encodes the transposase, which is important for its mobility (Britten, 1996). To accomplish transposition, ISs generally infect the target site to generate short direct repeats. Some ISs undergo transposition using a non-replicative or cut-and-paste mechanism, while others use a replicative copy-and-paste mechanism, where the first copy remains intact, while the second copy is used at the target site (Duval-Valentin et al., 2004).

The combination of different incompatibility (InC) groups of plasmids and MGEs help in the spread of ARGs. There is a strong interaction between conjugative plasmids and ISs. *In silico* analysis exhibited transfer network of about 250 groups, comprising nearly 60 ARG subtypes and 50 ISs connecting conjugative plasmids in genetically distinct pathogens (Che et al., 2021). In this analysis, IS*26*, IS*Ecp1*, and IS*6100* are the most predominant elements mediating the transfer of ARGs. IS*Aba125*-*bla*_NDM-1_, IS*Ecp1*/IS*26*-*bla*_CTX-M,_ IS*Apl1*-*mcr-1* are the ISs specifically involved in the transfer of New Delhi metallo-β-lactamase (NDM), extended-spectrum β-lactamase (ESBL) and mobilized colistin resistance (MCR)-encoding genes with different genetic backbones. Interspecies transfer mediated by IS*26* and IS*6100*, both belonging to the IS*6* family, was widely identified across many bacteria, involving about 20 genera belonging to 7 families (Cuzon et al., 2011).

Some of the ISs, specifically carry ESBL encoding genes. For e.g., homologous recombination mediated by IS*26* was found to be responsible for the spread of several variants of *bla*_NDM_ along with the other MDR encoding genes (Zhao et al., 2021). IS*Ecp1* belonging to the IS*1380* family is an effective mobilizer of *bla*_CTX-M_ by a unique transposition process using neighboring sequences by transposition. In several studies, IS*Ecp1*element was shown to be associated with *bla*_CTX-M-15_ and other *bla*_CTX_ alleles in both clinical and foodborne pathogens (Ranjbar et al., 2010; Shawa et al., 2021).

The genetic environment of the *mcr-1* structure indicated that this colistin resistance gene could be mobilized as an IS*Apl1*, flanked by the composite transposon (Tn*6330*). However, many *mcr-1* structure sequences have been identified without IS*Apl* or with a single-ended IS*Apl*, signifying its origin from the ancestral Tn*6330* by a copy-and-paste mechanism (Snesrud et al., 2018). In most of the bacteria, IS*Apl1* was identified either upstream or downstream of *mcr* with or without other ARGs (Anyanwu et al., 2020). A novel mobile resistance gene, *fexA* encoding resistance for florfenicol (a class of phenicol) has been detected both on the plasmid and chromosomes of *Campylobacter jejuni* (Tang et al., 2020a). The presence of IS*1216* around *fexA* appears to be important in the integration of the *fexA*-carrying gene segment along with *tet(L)-fexA-catA-tet(O)* gene array.

### Transposons

Transposons are a large and complex version of ISs with repetitive DNA sequences that can be transposed from one genome locus to the other (Siguier et al., 2006). This mobility can result in mutations, alter gene expression and induce chromosomal rearrangements. Transposons are directly involved in carrying the cargo genes such as ARGs, MGEs, ISs, and toxin-antitoxin modules (Babakhani and Oloomi, 2018). Conjugative transposons integrate into the DNA using different means of excision and integration compared to the classical transposons Tn*5* and Tn*10*. After excision, the conjugative transposons form a covalently closed circular intermediate that can either reintegrate in the genome of the same bacterial cell or transferred to other bacteria by conjugation (Salyers et al., 1995).

Transposon helps acquisition and spread of ARGs in several bacterial pathogens. IS*Chh1*-like transposon helps in acquiring MDR genes in *Campylobacter*, including the *optrA* gene that encodes an ATP-binding cassette F protein. The presence of *optrA* has confirmed its role in elevated minimum inhibitory concentration (MIC) to oxazolidinones and phenicols (Tang et al., 2020b). Interestingly, IS*Chh1*-like transposon also integrates with AMR genes such as *tet(O)*, *aphA3*, and *aadE-sat4-aphA3* gene cluster (Tang et al., 2020b). The function of IS*Apl1* transposon in the mobilization of plasmid-borne *mcr-*1 was first established in the mid-2000s. However, majority of the sequences reported subsequently had no IS*Apl1* (Wang et al., 2018a). This finding suggests that the IS*Apl1* transposon had been stabilized over time in the host’s genome background and is currently spreading *mcr-*1 through plasmid transfer (Wang et al., 2018a). The Tn*6330* transposon is responsible for the spread of *mcr-1* between various plasmids and chromosomes (Li et al., 2018). The association between plasmids and the transposon are further discussed under the section plasmids.

*Klebsiella pneumoniae* strains carry different type of transposons with several ARGs. In outbreak-associated *K. pneumoniae* strains, the β-lactam resistance encoding genes *bla*_TEM-1_ and *bla*_KPC-3_ had Tn*4401* element located upstream of the *bla*_KPC-3_ gene (Leavitt et al., 2010). A novel Tn*1696*-like composite transposon (designated as Tn*6404*) has been identified in a carbapenem-resistant isolate that carried *bla*_IMP-4_ and *bla*_SFO-1_ genes (Zhou et al., 2017). *aac(6’)-Ib, ant(3’)-Ia*, *bla*_TEM_ and *bla*_OXA-9_ genes were found be carried by the Tn*1331* (Tolmasky, 2000).

Several Tn*7*-like transposons have been identified to carry both an anti-MGE defense system and ARGs, indicating its multiple impacts on bacteria (Benler et al., 2021). In many pathogens, tetracycline resistance encoding *tet(M)* gene that spreads through Tn*916*-like elements carry *erm(B)* with or without the macrolide efflux genetic operon [*mef(E)-msr(D)*] (Marosevic et al., 2017). The conjugative transposon of the Tn*916*/Tn*1545* family carry several MDR determinants in Gram-positive pathogens. Most of these transposons uniformly harbor the *tetM* gene (Roberts and Mullany, 2011). An uncommon IS that has 84% homology with IS*Ec63* of Tn*3* family together with the *bla*_KPC_ gene and Tn*4401* fragments was found inserted in the *tra* operon of outbreak-associated enterobacterial isolates (Wozniak et al., 2021). These results indicate the important role of transposons, with stable integration into the target cell genome and the expression of AMR.

### Plasmids

Transfer of AMR conferring plasmids by conjugation is an another major factor involved in the dissemination of ARGs. Plasmid mobilization makes fitness costs in bacteria, which has been minimized through compensatory mutations. Association between plasmids and bacteria successfully shapes the progression of AMR. Normally, conjugative plasmids transfer AMR determinants using MGEs, including integrons, transposons, and ISs. Several factors that influence the rate of plasmid transfer, as well as its functional response have been identified. In addition, the toxin-antitoxin system (TAS) help in the maintenance of MDR plasmids that has been discussed in the section TAS.

**Figure 2:**
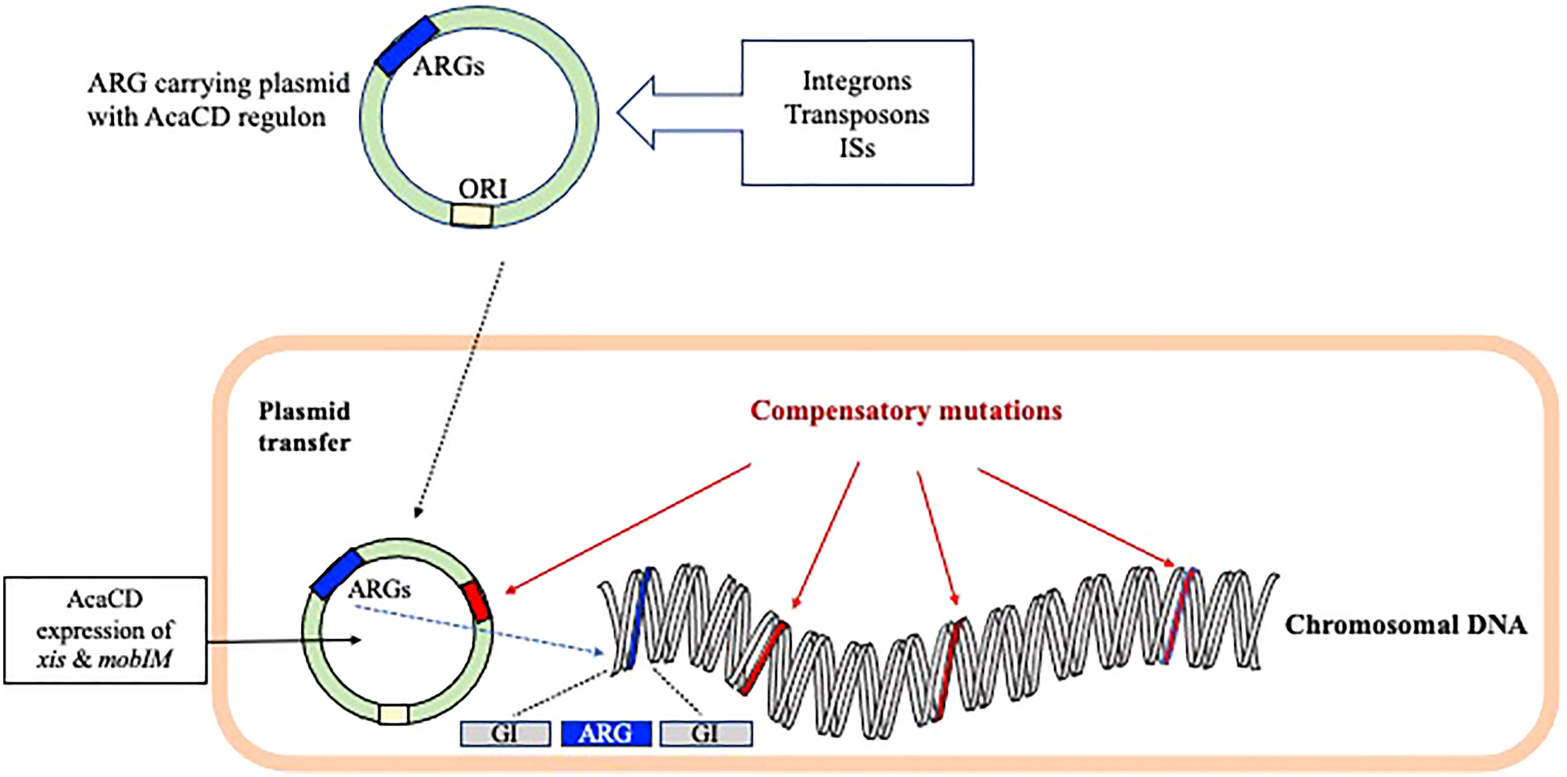
The role of plasmid and genomic island-encoded factors in the spread of AMR encoding genes. The mobility of selective Inc conjugative plasmid carrying the ARGs depends mostly on the master activator AcaCD regulon. The AcaCD supported expression of *xis* and *mobIM* helps in the excision and mobilization of AMR encoding genes, respectively. The fitness costs in bacteria has been minimized through plasmid/chromosomal compensatory mutations.

Plasmids are classified based on their incompatibility (Inc), as they have unique replication and partition systems. Plasmid and GI-encoded factors are important for the effective AMR-island excision, mobilization, integration, and regulation in the host bacteria. In some cases, the mobility of IncC or IncA/C conjugative plasmid depends on the transcriptional activation of multiple operons of the plasmid by the master activator AcaCD regulon (**Figure 2**). The regulatory network of AcaCD extends to the chromosomally integrated GIs, and express *xis* and *mobIM* for excision and mobilization, respectively (**Figure 2**) (Rivard et al., 2020). This regulon activates the expression of genes located in the SGI-1 and the MGI*Vch*Hai6, which is a GI, integrated in the *trmE* on chromosome I of *V. cholerae* non-O1/non-O139. Transfer of MGI*Vch*Hai6 confers resistance to β-lactams, sulfamethoxazole, tetracycline, chloramphenicol, trimethoprim, and streptomycin/spectinomycin (Carraro et al., 2016).

**Figure 3:**
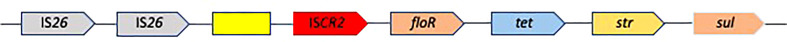
An IncA/C plasmid with defined AMR gene cluster. The basic structure of the IncA/C plasmid carrying the *floR-tetAR-strAB* cluster is with three integration hotspots with two IS*26*, IS*CR2* and an unknown ORF (yellow box) in between that facilitate the acquisition of additional genes.

IncA/C plasmid in various bacterial pathogens shares a conserved backbone with several MGEs with ARGs. The IncA/C plasmid with *bla*_CMY-2_ represents a unique lineage that has been reported in a diverse group of bacteria. Mostly, the basic structure of this plasmid includes a *sul2* module containing the *floR-tetAR-strAB* cluster and a Tn*21*-like element (Fernández-Alarcón et al., 2011). As shown in **Figure 3**, this broad host range lineage is comprised of three integration hotspots facilitating the acquisition of additional genes through integrons, IS*26*, IS*Ecp1* and IS*CR2* elements (Suzuki et al., 2010). IS*CR*2 plays an important role in mobilizing the *floR-tetA-strAB-sul2* cluster. In IncA/C plasmid lineage, the *sul2* module is steadily maintained, whereas the *bla*_CMY-2_ and Tn*21*-like regions vary among different species. IS*Ecp1* existed in all the copies of IncA/C-encoded *bla*_CMY-2_ and was found to be involved in ‘one-ended transposition’ for self-mobilization along with the other ARGs (Toleman and Walsh, 2011). Transfer and replication of β-lactamase genes mediated by IS*Ecp1*-like elements have been identified in the *tra1* region on IncA/C plasmids of many bacterial species (D'Andrea et al., 2011). Plasmids with *tra* gene contain several ORFs that encode important proteins needed for effective conjugation.

Transmission of the *bla*_NDM-1_ gene in *E. coli* of animal origin is enhanced due to the presence of IncFII plasmids. IncFII plasmid with *bla*_NDM-1_ is stably maintained in the animal gut microbiome even in the absence of carbapenem selection pressure (Lin et al., 2016). The spread of frequently reported ESBL-encoding *bla*_CTX-M-15_ appears to be supported by the presence of the IncF plasmid in *E. coli* that contains a Tn*2*-*bla*_TEM-1_ transposon (Branger et al., 2018). IncX3 plasmid was shown to be important in the emergence and spread of *bla*_NDM-5_, which is associated with resistance to most of the β-lactams (Ma et al., 2020). The transconjugants showed enhanced growth and biofilm formation. This additional fitness might be one of the reasons for the global dissemination of IncX3 plasmid with *bla*_NDM-5_.

Frequently identified genes like *mcr* are located on the broad-host-range plasmids and their proximity to different ISs helps in the effective transmission between different bacterial species. In a bioinformatic analysis, interspecies transfer of AMR was found mediated mainly by the conjugative plasmids having ISs belonging to the IS*6* family, *i.e.*, IS*26* and IS*6100* (Che et al., 2021). However, such transfers are governed by multiple factors, including the genetic background of the ARGs, the fitness cost of the host, etc. Plasmid-mediated colistin resistance is prompted by phosphoethanolamine transferases encoding *mcr* variants that are localized on the IncX4 type plasmid. MCR-1 imposes a fitness cost to its host bacterium and hence its spread requires a high plasmid conjugation frequency. With these features, IncI2, IncX4, and IncH12 plasmids are epidemiologically successful genetic vectors that spread *mcr-1* globally (Vázquez et al., 2022). In *E. coli*, the transcriptional regulator *pixR* increases plasmid transmissibility, invasion, and persistence of *mcr-1*-bearing plasmids (Yi et al., 2022). Transmission of some of the *mcr* alleles from bacterial chromosomes to MGEs seems to occur as an independent event. For e.g., a novel variant *mcr-5* has been identified in colistin-resistant *S. enterica* Paratyphi B isolates that harbored ColE-type plasmids containing transposon of the Tn*3* family (Borowiak et al., 2017). An uncommon Tn*As3*-like transposon was detected on a self-transmissible IncP plasmid carried unrelated AGRs, *bla*_NDM-5_ and *mcr-3* in MDR clinical isolates of *E. coli* (Liu et al., 2017).

The hybrid plasmid of both type-1 and type-2 IncC has been detected in *Vibrio alginolyticus*, which displayed resistance to almost all the β-lactam antibiotics and also had a novel carbapenemase ‘*Vibrio* metallo-β-lactamase-1’ (Zheng et al., 2020). The chloramphenicol-florfenicol resistance gene (*cfr*) and its alleles that encode an rRNA methyltransferase (*ermE*), has been detected in many Gram-positive and Gram-negative pathogens (Tang et al., 2017). The *cfr* alleles generally confer resistance to phenicols, lincosamides, oxazolidinones, pleuromutilins, and streptogramin-A. The *cfrC* allele was detected on transferable plasmids flanked by two copies of IS*26*. Through homologous recombination, *cfrC* can loop out the intervening sequences (Deng et al., 2014). Transmission of high-level aminoglycoside resistance (16S-rRNA methylase, *rmtB*) and a quinolone efflux pump (*qepA*) in *E. coli* are associated with Tn*3*, IS*26*, and IS*CR3* in an IncFII plasmid (Deng et al., 2011). Plasmid-mediated *qepA2* and multiple chromosomally-mediated fluoroquinolone resistance determinants also increases fluoroquinolone resistance to several folds (Machuca et al., 2015). The presence of *qepA2* induces survival cost in *E. coli*, but it was counterbalanced by the deletion of multiple antibiotic resistance (*marR*) gene (Machuca et al., 2015).

Integrative plasmids play an important role in the stability and spread of virulence and ARGs. *S.* Enteritidis-specific virulence plasmid, pSEN was found to be integrated into an IncHI2 MDR plasmid having the cephalosporin and fosfomycin resistance determinants *bla*_CTX-M-14_ and *fosA3*, respectively (Wong et al., 2017). The replicative transposition process has been mediated by IS*26*, which is usually identified in many MDR plasmids. In *S.* Typhimurium, IncHI2-type plasmid harboring the olaquindox/quinolone AB-encoding gene *oqxAB* is responsible for the ciprofloxacin resistance (Lian et al., 2019). After acquiring this plasmid, the chromosomal efflux pump genes *acrAB*, *tolC*, and *yceE* remain upregulated and maintained the survival of ciprofloxacin exposed *S.* Typhimurium.

Epigenetic compatibility of quinolone resistance (Qnr) determinants in the host genome depends on the bacterial species and other factors. Plasmids carrying the QnrA determinant are stable in *E. coli*, whereas the SmQnr, which was present in a Gram-negative, multidrug resistant, opportunistic pathogen *Stenotrophomonas maltophilia*, was found to be unstable despite both the proteins exhibiting homologous tertiary structures (Sánchez and Martínez, 2012). This mechanism signifies that the fitness costs associated with the acquisition of SmQnr may not be derived from the metabolic burden, but the acquired gene seems to be important in initiating specific changes in the host bacterial metabolic and regulatory network. The plasmid-mediated quinolone resistance determinant OqxAB-mediates resistance to many antimicrobials such as chloramphenicol, quinolones, quinoxalines, trimethoprim, etc., mostly among the members of the family Enterobacteriaceae. *oqxAB* flanked by IS*26* elements forms a composite transposon Tn*6010*, which has been detected mostly in the IncHI2 plasmid (Ruiz et al., 2012; Wang et al., 2017; Li et al., 2019). This *oqxAB* has been clustered with several other resistance genes on the same plasmid, including *aac(6’)-Ib-cr*, *qnrS*, *bla*_CTX-M-55_, *rmtB*, *fosA3*, and *floR* (Wang et al., 2017).

### Phages

Recent findings suggest that phage-bacterial association play a substantial role in the transmission of AMR, especially in bacteria from the environment and foods of animal origin. Metagenomic analysis of food animals, natural water bodies, effluents, and soil supplemented with animal manure supported the view on the spread of ARGs through bacteriophage DNA and prophage elements (Ross and Topp, 2015; Shousha et al., 2015; Mohan Raj and Karunasagar, 2019; Balcázar, 2020). ARGs containing extracellular DNA is protected from the action of DNase, as they are packed in the phage’s capsid proteins. These DNA fragments can be safely transferred to bacterial cells, following their integration into the specific regions of the chromosome through RecA (essential for the repair and maintenance of DNA)-dependent homologous recombination. The prophage-containing bacteria can further transfer the ARGs by HGT. Gaining prophage influences bacterial fitness through the transfer of several genes, including ARGs. Under *in vitro* conditions, it was demonstrated that bacteria confer fitness benefits by carrying prophage-encoded ARGs (Haaber et al., 2016; Wachino et al., 2019; Wendling et al., 2021). Findings of *in silico* analysis showed that the role of polyvalent-bacteriophages, that are capable of infecting more than one host, is important in the intergeneric transmission of ARGs that encode an ESBL (*bla*_CTX-M_), ABC-type efflux permease (*mel*), and ribosomal protection protein (*tetM*) loci (Gabashvili et al., 2020).

Analysis of more than thirty viromes from human and non-human sources (animals and environment) indicated that the latter group was a reservoir of ARGs (Lekunberri et al., 2017). In chicken feces and water samples, bacteriophage genomes carried several important ARGs (Yang et al., 2020; Zare et al., 2021). Moreover, several copies of ARGs, including β-lactam, aminoglycoside, and fluoroquinolone resistance have been detected in the DNA of *E. coli* phage YZ1 (Wang et al., 2018b). Enterotoxigenic *E. coli* prophage carried several ARGs, which expressed resistance to sulfamethoxazole-trimethoprim, chloramphenicol, tetracycline, aminoglycoside and narrow-spectrum β-lactamase (*bla*_TEM-1b_). This MDR transmitting prophage had Tn*2* transposon with serine recombinase gene flanking the *bla*_TEM-1b_ and the IntI1 together with the Tn*As2* transposon (Wang et al., 2020a). In *E. coli* isolates of animal origin, phage-like plasmid with-*mcr1* excised and formed a circular intermediate before integration into plasmids containing the IS*Apl1* element (Li et al., 2017a). This event may be more complex upon translocation into phage-like vectors, which can be transmitted *via* transduction events.

## Efflux pump system

**Figure 4:**
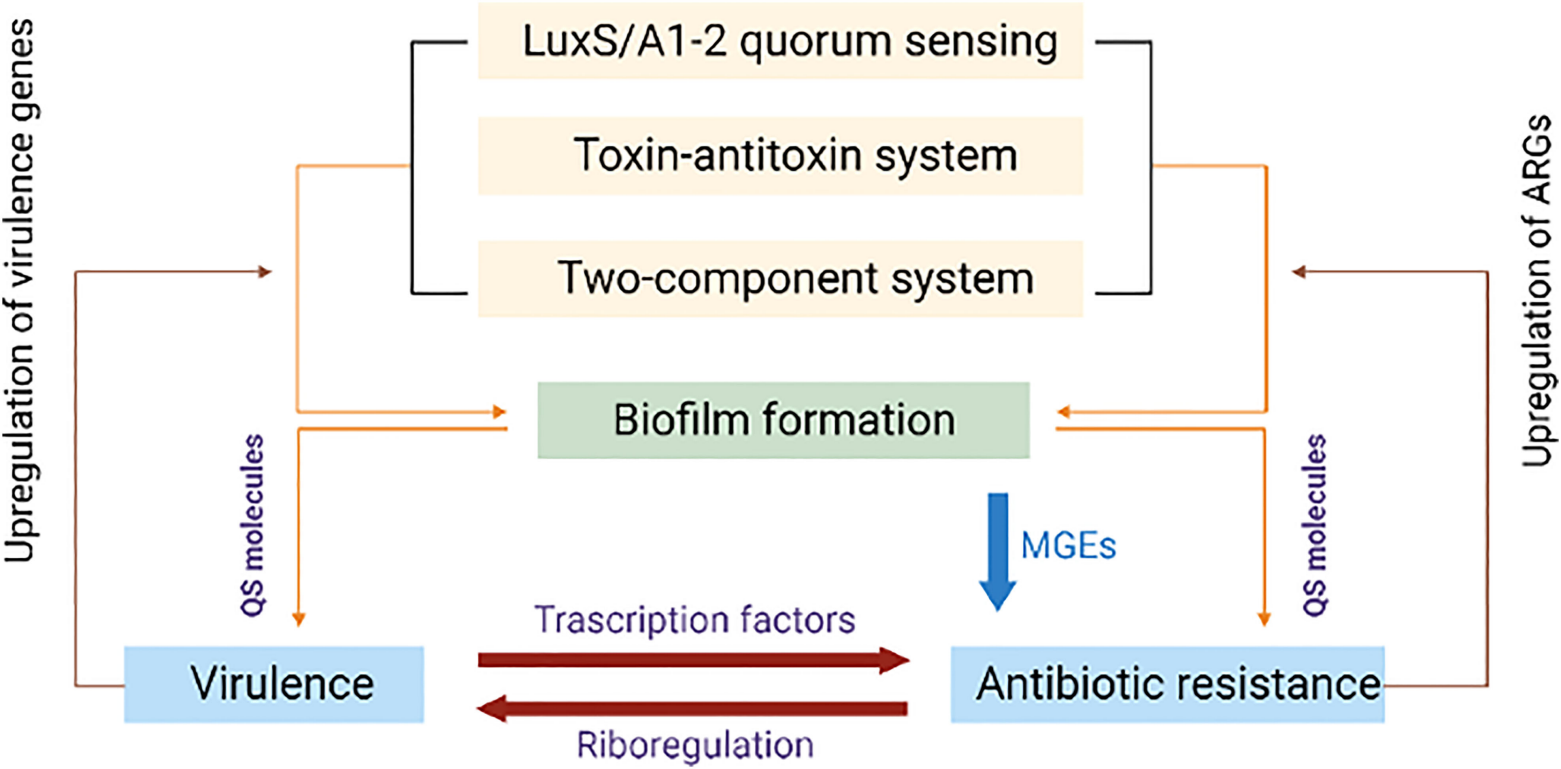
Diagrammatic representation of shared regulatory network mechanisms involving antibiotic resistance and virulence. Quorum sensing (QS), toxin-antitoxin (TAS) and two component systems (TCS) upregulate the biofilm formation encoding genes that successively upregulate quorum sensing molecules. These molecules influence antibiotic resistance genes and virulence. Up-regulated virulence and antibiotic resistance genes consequently upregulate QS, TAS and TCS.

Efflux pumps are a mechanism of an advanced defense system in bacteria. Efflux pump systems (EPS) can force out antibiotics from the bacterial cell to maintain the antibiotic concentration below the lethal threshold inside the cell. Efflux pumps can confer resistance to a single or a structurally diverse class of antibiotics. The distribution of efflux pump genes and the upregulation of their expression determines the extent of resistance to many antimicrobial agents. Generally, chromosomes encode several MDR efflux pumps and their expression is controlled by point mutations in the regulatory genes (Nowak et al., 2015). Efflux pumps can be classified into several categories based on their structure, the number of transmembrane spanning regions, energy sources, and substrates. Accordingly, the major subfamilies of efflux pumps include, i) Resistance Nodulation Division (RND) family, ii) Major Facilitator Superfamily (MFS), iii) ATP (adenosine triphosphate)-binding cassette (ABC) superfamily, iv) Small Multidrug Resistance family and v) Multidrug and Toxic compound extrusion family. Several coordinated networks of efflux transporters have been identified in bacteria. Complex signaling pathways are involved in the EPS-mediated resistance mechanisms to protect bacteria (**Figure 4**).

RND is the major mechanism of MDR in Gram-negative bacteria. This larger efflux transporter is an inner membrane protein that networks with a periplasmic fusion protein and an outer membrane channel protein to form a tripartite complex to directly export antibiotics from the bacterial cells. Members of Enterobacteriaceae use AcrAB-TolC and OqxAB-TolC efflux pumps to resist the action of fluoroquinolones, β-lactams, tetracyclines, macrolides, oxazolidinone, quinolones, chloramphenicol (Swick et al., 2011). A three-gene operon *cmeABC* is the major EPS in *Campylobacter* and plays a key role in mediating resistance to structurally dissimilar antimicrobials. The multidrug efflux CmeDEF interacts with CmeABC and increases the antimicrobial resistance and the cell viability (Akiba et al., 2006). In *C. jejuni*, CmeC functions as a multidrug efflux transporter, and overexpression of the *cmeGH* operon significantly increases its resistance mostly to fluoroquinolones as well as exogenous hydrogen peroxide (Jeon et al., 2011). The tripartite protein components of RND are post-translationally changed to N-linked glycans in *C. jejuni*. The multifunctional role of *N-*linked glycans stabilizes the protein complexes, by improving their thermostability and increasing their propensity for protein-protein interaction, which in turn facilitates AMR through multidrug efflux pump activity (Abouelhadid et al., 2020).

Involvement of the other RND-type efflux systems such as AdeABC, AdeFGH, and AdeIJK are important for intrinsic MDR in several pathogens. Overexpression of AdeABC and AdeFGH systems in *Acinetobacter baumannii* induces resistance to aminoglycosides, β- lactams, chloramphenicol, fluoroquinolones, macrolides and tetracyclines (Yoon et al., 2015). AdeABC also alters membrane-associated cellular functions such as the formation of biofilm and plasmid transfer. Multi-efflux pumps simultaneously protect the pathogens against several antimicrobials. In the three multi-drug efflux pump systems (MacAB-TolC [ABC efflux], MFS drug transport system, and AcrAB-TolC) of *S.* Typhi, *tolC*, *macB*, *acrA*, *acrB*, and *mdfA* are involved in multiple resistance pathways that protect the pathogen from macrolides, chloramphenicol, tetracycline, novobiocin, quinolones, fluoroquinolones and β-lactams (Debroy et al., 2020). The genes related to tripartite efflux pumps *mdtEF-tolC* and the ABC family efflux pump *macAB-tolC* are important in Shiga toxin-producing *E. coli* (STEC) O157:H7 (Miryala and Ramaiah, 2019). About 20 genes are involved in multi-efflux pumps in this pathogen, which are directly or indirectly associated with the MDR.

## Toxin-antitoxin system

Toxin-antitoxin system (TAS) present in the mobile genetic scaffolds consists of a stable toxin component that targets an essential cellular process and an antitoxin that counteracts the activity of the toxin. Recent findings suggest that the TAS are mostly used to lower bacterial metabolism during stress, prevent the invasion of phages, stabilize genetic elements, and support biofilm formation (Song and Wood, 2020). Depending on the molecular nature and their mode of interaction with the toxins, TAS is classified into several types. Importantly, in type I TAS, toxin and antitoxin are protein and RNA, respectively, but in the type II TAS, both toxin and antitoxin are proteins that may differ greatly among several bacterial species. TAS can either be on a plasmid or can be chromosomally encoded, which is generally associated with bacterial stress adaptation.

In vancomycin- resistant enterococci (VRE), specific TA pair, the *mazEF* system located on plasmids is responsible for the resistance to most major classes of antibiotics (Moritz and Hergenrother, 2006). In addition, the plasmid replicon types of pRE25, pRUM, pIP501, and pHT-β in VRE are known to be linked to glycopeptide resistance and stabilizing TA systems. Most of the VRE strains belonged to these plasmid replicon types assigned to the clonal complex 17 (Rosvoll et al., 2010). The SplTA is widely present in plasmids harboring the carbapenem resistance gene that helps in the maintenance of plasmids and provide stability to the transferred genetic elements. The SplTA and HigBA are the most prevalent plasmid-associated TAS found in carbapenem resistant *A. baumannii* (Jurenaite et al., 2013). Cross-resistance to chlorhexidine-colistin has also been reported in carbapenemase-producing *K. pneumoniae* that has the type II TAS (Bleriot et al., 2020). In *V. cholerae*, the *mosAT* encoded TAS present within the ICE-SXT (conferring resistance to sulfamethoxazole and trimethoprim) element helps to maintain sulfamethoxazole-trimethoprim resistance (Wozniak and Waldor, 2009). In *Staphylococcus aureus* TAS, *mazEF* encodes the RNase MazF and the antitoxin MazE. This gene cluster enhances tolerance to several antimicrobials (Ma et al., 2019a).

In *E. coli* and *Salmonella*, ParDE type II TAS that exists in Inc (I and IncF-type) plasmids not only helps in the plasmid stability, stress response, and biofilm formation, but also supports genes encoding resistance to aminoglycoside, quinolone, and β-lactams (Kamruzzaman and Iredell, 2019). IncX plasmids are important in the transmission of carbapenem and colistin resistance. Sequence analysis of IncX plasmids indicates the existence of RelE/ParE toxin superfamily within the IncX1 and IncX4 subgroups (Bustamante and Iredell, 2017). In MDR IncC plasmids, the TAS functions as an effective addiction module and maintains plasmid stability even in an antibiotic-free environment (Qi et al., 2021). In *K. pneumoniae*, an IncHI2 plasmid has been linked with HipBA and RelBE TAS, which helps the plasmid to maintain multiple ARGs, including *catA2, aac(6’)-Ib, strB, strA, dfrA19, bla*_TEM-1_ *, bla*_SHV-12_ *, sul1, qacEΔ1, ereA, arr2*, and *aac3* along with several other genes encoding resistance to heavy metals (Zhai et al., 2016).

## Influence of small RNAs network

**Figure 5:**
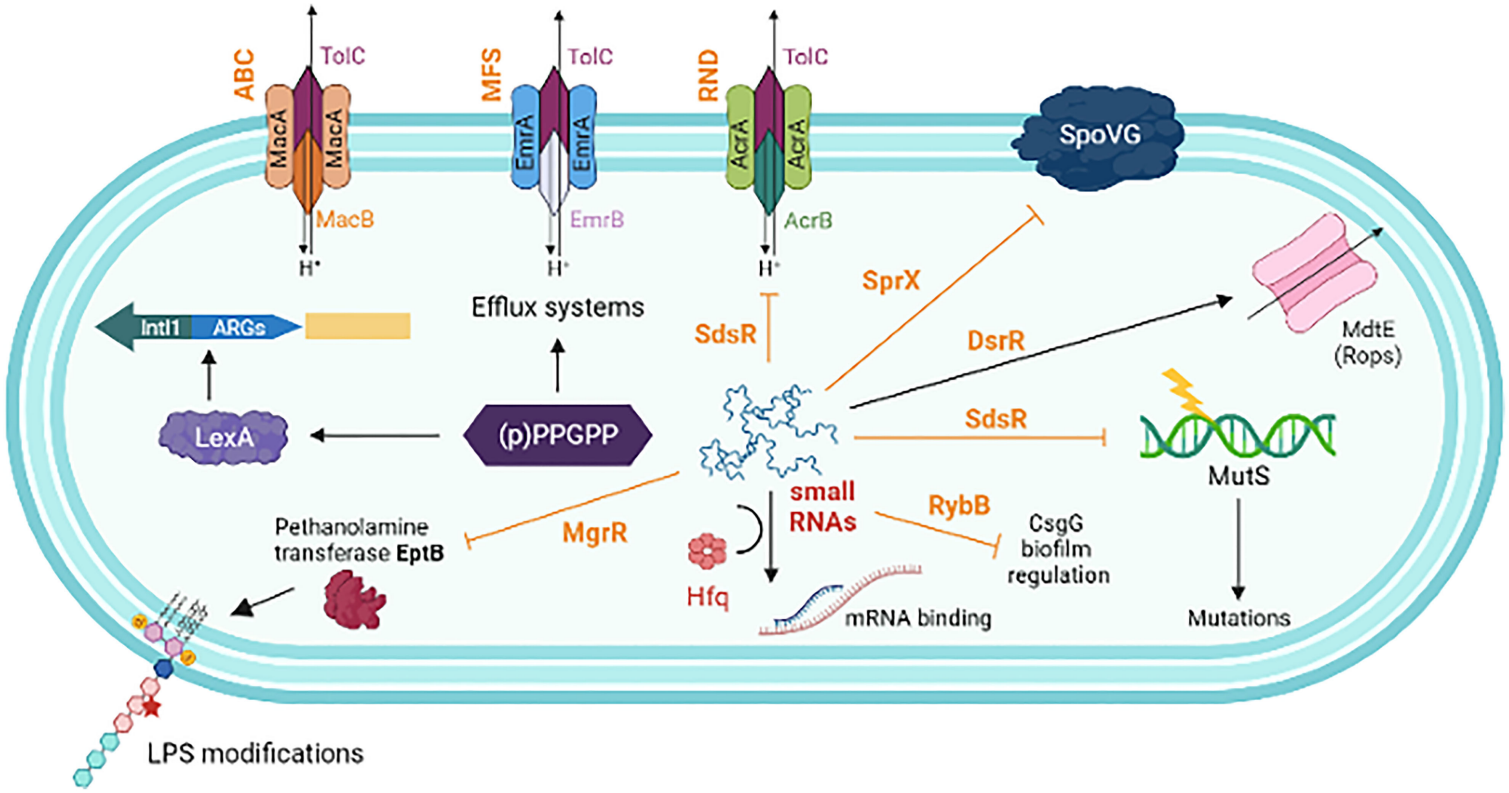
A schematic representation showing the interplay of different factors influence in antimicrobial resistance. (p)ppGpp increases the efficiency of HGT, AMR cassette acquisition through *IntlI* and upregulates the expression of various components of the efflux pumps. Non-coding small RNAs (sRNAs) play a major role in post-transcriptional regulation of gene expression. This includes, negatively regulated targets of MgrR involved in LPS modification (sensitivity to Polymyxin B); SdsR, repress the expression of *tolC*, the gene encoding the OMP of many multidrug resistance efflux pumps; SdsR also base-pair with *mutS* mRNA to repair the DNA after exposure to β-lactams; SprX (a.k.a. RsaOR) influence resistance to glycopeptides by downregulating the SpoVG; DsrA activates the expression of MdtE, which increases efflux system to antibiotic such as oxacillin, cloxacillin, erythromycin, novobiocin etc. RybB negatively influences the expression of *csgD* transcription, which is the master regulator of biofilm formation. sRNAs strongly interact with the co-factor Hfq, which enhances sRNA stability and facilitates base-pairing of sRNAs with multiple target mRNAs. Small RNAs are typed in dark brown. Solid arrows indicate activating interactions and T-arrows indicate inhibiting interactions.

Regulatory small RNAs (sRNA) are important in stabilizing the configuration of the bacterial envelope and uptake of antimicrobials by controlling porins and transporters at the cell surface. The sRNA network that controls several functions related to AMR is shown in **Figure 5**. The post-transcriptional network of sRNA communication is employed to identify the network centers and regulatory functions (Mediati et al., 2021). Though the sRNAs amply exists within the MGEs, their direct involvement in the regulation of ARGs has not been studied in detail. The contribution of sRNAs to intrinsic resistance has been identified in horizontally acquired ARGs. However, their AMR-related functions are complex in different bacteria. The sRNA MicF-mediated repression of outer-membrane porin OmpF in *E. coli* reduces membrane permeability to inhibit cephalosporin, norfloxacin, and minocycline uptake (Cohen et al., 1988). *S. aureus* sRNA, SprX specifically downregulates stage-V sporulation protein-G, and SpoVG, which increases resistance to glycopeptides (Eyraud et al., 2014). Over-expression of some of the sRNAs (e.g., Sr0161, Sr006) in *P. aeruginosa* increased resistance to meropenem and polymyxin-B (Zhang et al., 2017). *A. baumannii* fosfomycin efflux (AbaF) is one of the primary targets of AbsR2*5*, which negatively regulates the major facilitator superfamily efflux pump gene *abaF* (Sharma et al., 2017). Interruption of this *abaF* indicates that it contributes to fosfomycin susceptibility, reduction in biofilm formation, and virulence. SdsR is one of the highly conserved enterobacterial sRNAs. In *S. sonnei*, SdsR promotes resistance to fluoroquinolone by regulating the expression of an efflux pump TolC (Gan and Tan, 2019).

## Guanosine pentaphosphate/tetraphosphate signaling

An alarmone, guanosine pentaphosphate or tetraphosphate ([p]ppGpp) is the effector molecule, which is involved in the up-regulation of several genes involved in the bacterial response to extracellular stress, including AMR. (p)ppGpp-mediated stringent response can support microbes to survive even in the absence of specific resistance genes due to complex modulation network activities involving several targets/proteins and other small signaling molecules (Das and Bhadra, 2020). The *relA* gene catalyses the synthesis of (p)ppGpp during antibiotic or other stresses. In *P. aeruginosa*, mutations in the *spoT* and *dksA* (involved with the stringent response) induce higher levels of intracellular ppGpp concentration, which supports tolerance to quinolones (Viducic et al., 2006). In addition, (p)ppGpp helps in HGT, including the integron-mediated acquisition of AMR gene cassettes (Strugeon et al., 2016). In several pathogens, (p)ppGpp mediates resistance to vancomycin (*Enterococcus faecalis*), aminoglycoside (*Salmonella*), oxacillin (*S. aureus*), tetracycline and erythromycin (*V. cholerae*) (Abranches et al., 2009; Koskiniemi et al., 2011; Mwangi et al., 2013; Kim et al., 2018).

## Quorum sensing network

The intercellular cell to cell communication mechanism through quorum-sensing (QS) controls the expression of several genes through signaling molecules called autoinducers, which plays a significant role in the adaptation and survival of bacteria. Regulation of the LuxS/AI-2 QS system has been identified in many bacterial genomes (Waters and Bassler, 2005). One of its functions is AMR that include quinolones (inhibition of DNA replication by complexing with DNA and DNA gyrase and/or topoisomerase IV), sulfonamides (prevention of tetrahydrofolate synthesis by inhibiting dihydrofolate reductase and dihydropteroate synthetase), tetracyclines (preventing aminoacyl-tRNA binding to the ribosomal-A site), β-lactams (preventing transpeptidation) and glycopeptides (preventing transpeptidation) (Wang et al., 2019). In addition, LuxS/AI-2 affects drug resistance through biofilm formation, MGEs, efflux pumps, VraSR-TCS, folate synthesis pathway, etc (Wang et al., 2019).

## Co-factors and co-selection

In bacteria, many co-factors modulate the activity of AMR. Several findings now describe about the presence of chemicals, especially the heavy metal contaminations that enhance the AMR through co-selection. This section highlights the role of co-factors and co-selection in the development and dissemination of AMR.

The *β*-lactam antibiotics interfere with cell-wall biosynthesis by inactivating penicillin-binding proteins (PBPs), which are important for bacterial survival. The interaction between cell-wall integrity and the resistance mechanisms help in deactivation of cell-wall-targeting antibiotics through cell-wall recycling pathway. Several Gram-negative bacteria use DNA-binding proteins for ampicillin resistance such as the LysR-type transcriptional regulator (AmpR) system to mobilize resistance mechanisms in response to *β*-lactams by changing the composition of the cell wall-associated peptidoglycan (Dik et al., 2018). The AmpR system also releases GlcNAc-anhydro-MurNAc-oligopeptides into the periplasm and the muropeptides are transported into the cytosol through an *ampG*-encoded AmpG transporter (Chahboune et al., 2005). Further, the activation of the *amp* genes marks the expression of AmpC β-lactamase because of its cognate regulator AmpR, which regulates MDR (Dhar et al., 2018). In addition to carbapenemases, overexpression of AmpC, efflux pumps, or porin loss might contribute to the carbapenem resistance in pathogens like *K. aerogenes* (D'Souza et al., 2021). The network of AmpC β-lactamase overexpression has been recognized due to AmpG in conferring resistance to β-lactam antibiotics, including cephalosporins and carbapenems. Transposon insertion mutations in the AmpG β-lactamase expression pathway make the *Klebsiella* strains susceptible to extended- and broad-spectrum cephalosporins (D'Souza et al., 2021).

CpxAR is commonly present in Gram-negative bacteria, which sense and regulate osmotic pressure and also confer AMR. In the absence of the AcrB efflux pump, the CpxR is overexpressed, which lead to resistance to β-lactam antibiotics. Thus, modifications in the expression of outer membrane proteins (OMPs) can play an important role in acquiring resistance to aminoglycosides and β-lactams (Hu et al., 2011). Phosphoethanolamine (PEtN) transferases (*eptA* and *eptB*) confer one of the mechanisms for colistin resistance. *eptA* catalyzes the transfer of PEtN from phosphatidylethanolamine onto the lipid A of LPS and *eptB* catalyzes the addition of a PEtN moiety to the outer 3-deoxy-d-manno-octulosonic acid (Kdo) residue of a Kdo(2)-lipid A. Presence of *mcr*-*1* supplements the activity of *eptB* gene in higher transcriptional expression when the *E. coli* exposed to a sub-inhibitory concentration of colistin (Elizabeth et al., 2022).

Several other cofactors have been identified for promoting MDR. Collateral-resistance/allogenous selection occurs when an antibiotic induces mutation(s) that express resistance to the second antibiotic. This form of selection has been described in aminoglycosides, β-lactams, macrolides, tetracyclines, and quinolones/fluoroquinolones (Baquero et al., 2021). Many other interactions between antibiotics and biocides/heavy metals, may have co-selective or counter-selective consequences on AMR (Gilbert and McBain, 2003; Ciric et al., 2011; Rensch et al., 2013). The formation of complexes between metal cations and certain antibiotics and metal-dependent efflux induces resistance to β-lactam and tetracycline (Wales and Davies, 2015). Some of the important non-antimicrobial agents that induce cross-antimicrobial resistance among pathogenic bacteria is shown in **Table 1**.

**Table 1:** Components of non-antimicrobial agents that induce cross-antimicrobial resistance among pathogenic bacteria.

Non-antimicrobial agents	Antimicrobial resistance	Pathogen	Mechanism	Reference
As, Cd, Cr, Cu, Hg, Ni, Pb	Aminoglycoside, Amphenicol, Cephalosporin, Penicillin, Quinolone, Tetracycline, Sulphamethoxazole-trimethoprim	*E. coli, P. aeruginosa*	Outer membrane proteins	Choudhury and Kumar, 1996
Co	Tetracycline	*Bacillus subtilis*	Efflux pumps	Cheng et al., 1996
Ag	Cephalosporins	*E. coli*	Outer membrane proteins, efflux pumps	Li et al., 1997
Cd, Zn	β-lactams, Erythromycin, Kanamycin, Novobiocin, Ofloxacin	*Burkholderia cepacia*	DsbA–DsbB multidrug efflux system	Hayashi et al., 2000
Cd, Co, Zn	Clindamycin, Erythromycin, Josamycin	*L. monocytogenes*	Multidrug efflux system	Mata et al., 2000
V	β-lactams, Chloramphenicol, Fluoroquinolones, Tetracyclines, Ticarcillin, Clavulanic acid	*P. aeruginosa*	MexGHI–OpmD efflux pump, RND efflux pumps	Aendekerk et al., 2002
BKC	Amikacin, Tobramycin	*P. aeruginosa*	Efflux pumps	Joynson et al., 2002
As, Cr, Cu, Hg, Ni	β-lactams, Novobiocin	*S.* Typhimurium	Multidrug efflux system	Nishino et al., 2007
BKC	Ciprofloxacin, Gentamicin	*L. monocytogenes*	Efflux pumps	Rakic-Martinez et al., 2011
Cu	Erythromycin, Tetracycline, Vancomycin	*Enterococcus* spp.	Multidrug efflux system	Hasman and Aarestrup, 2002; Agga et al., 2014
TCS	Enrofloxacin, Sulphamethoxazole-trimethoprim	*S.* Typhimurium	Efflux pumps	Gantzhorn et al., 2015
Ag, Cu	Aminoglycosides, Macrolides, Tetracyclines, Trimethoprim	*S.* Typhimurium	Efflux pumps	Mourão et al., 2015
As, Cr, Cu, Hg, Ni	Chloramphenicol, Cefotaxime Penicillin, Tetracyclines,	*S.* Typhimurium, *P. aeruginosa*	Multidrug efflux system	Teixeira et al., 2016
Cd	Ampicillin, Chloramphenicol, Ceftizoxime, Ciprofloxacin	*S.* Typhi	Multidrug efflux system	Kaur et al., 2021

Ag, silver; As, arsenic; BKC, benzalkonium chloride; Cd, cadmium; Co, cobalt; Cr, chromium; Cu, copper; Hg, Mercury; Ni, nickel; Pb, lead; TCS, Triclosan; V, vanadium; Zn, Zinc.

The global repressor, histone-like nucleoid-structuring protein (H-NS) is a key transcriptional repressor of horizontally transferred genes in many bacteria. Upregulation and increased expression of genes associated with aminoglycosides, β-lactams, chloramphenicol, colistin, sulfonamides, trimethoprim and quinolones resistance are modulated by the H-NS (Rodgers et al., 2021). In *A. baumannii*, H-NS regulates the expression of genes involved in pathogenesis and resistance to colistin. The *Δhns* mutant confirms the role of H-NS as a transcriptional regulator, ameliorating the *A. baumannii* transcriptome associated with AMR.

The SOS response is a conserved regulatory network in bacteria that has been induced in response to DNA damage. SOS response is associated with AMR pathogens. In methicillin-resistant *S. aureus* (MRSA), oxacillin is one of the stimulating factors of a β-lactam-mediated SOS response through *lexA/recA* regulators that leads to the increased mutation and development of resistance. PBP1 also plays an important role in SOS-mediated *recA* activation and resistance selection in MRSA (Plata et al., 2013). Functional analysis showed that PBP1 depletion abolishes both β-lactam-induced *recA* expression/activation and increased mutation rates during resistance selection. Mutations enhancing the AMR involve fitness costs. However, the specific molecular mechanism of resistance in the bacterial fitness cost has not been investigated in detail. In *P. aeruginosa*, it was shown that the impact of mutations in the RNA polymerase gene *rpoB* influences the fitness cost of rifampin resistance (Qi et al., 2014). This modification occurs due to alteration in the relative transcript levels of essential genes that defend against the reduced RNA polymerase activity.

Bacteria recurrently encounter metals in their living environment, which can either be beneficial or harmful. The bacteria can use some of the metals as essential micronutrients. Co-selection of heavy metals and AMR exits as an overlapping mechanism of physiological intersection (cross-resistance) or genetic interrelation (co-regulation/co-resistance). An increase in AMR is associated in part with the co-selection of resistance to heavy metals and biocides/disinfectants used in livestock farming. These anthropogenic or non-antimicrobial agents can induce bacterial adaptations that cause decreased susceptibility or resistance to one or more antimicrobials even in the absence of any selection pressure (**Table 1**). Several cellular mechanisms are established to support the selection of genetic determinants associated with AMR. When *E. coli* strains were exposed to chlorhexidine digluconate, an increase in resistance to ampicillin, amoxicillin/clavulanic acid, cefoxitin, cefpodoxime, and cephalothin has been reported due to an increase in the RND efflux pump activity and upregulation of genes involved in cell metabolism (Wesgate et al., 2020).

Presence of plasmid-borne copper resistance genes *tcrB* and *pcoD* are linked with erythromycin, vancomycin, and tetracycline resistance with the respective encoding genes *ermB, vanA*, and *tetB* in human and animal pathogens (Hasman and Aarestrup, 2002; Agga et al., 2014). Copper efflux-associated *tcrB* and erythromycin resistance *erm*(B) genes were transferred by conjugation from the environmental *Enterococcus hirae* to *E. faecalis* (Pasquaroli et al., 2014). Intake of copper has some influence on AMR. In *E. faecalis* isolated from copper-fed pigs, chromosomally located *tcrYAZB* operon, *tetM*, and *vanA* are identified for copper, tetracycline, and vancomycin resistance (Zhang et al., 2015a). In livestock associated MRSA, genes encoding resistance to macrolides (*ermT*), tetracyclines (*tetL*) aminoglycosides (*aadT*), and trimethoprim (*dfrK*) are co-located together with *copA*, *cadDX*, and *mco*, which encodes resistance to copper and cadmium transporters and a copper oxidase, respectively (Gómez-Sanz et al., 2013). A similar mechanism was also reported in MDR *Salmonella* with an efflux mechanism for copper and silver through a chromosomally located *sil* operon (Mourão et al., 2015).

Reduced susceptibility to benzalkonium chloride in *Listeria monocytogenes* has been attributed to the presence of an EPS and linked to the reduced susceptibility to ciprofloxacin and gentamicin (Rakic-Martinez et al., 2011). Disinfectants like triclosan enhance AMR in many foodborne pathogens. Genetic changes in *fabI*, *rpoS*, and *rpoD* have been associated with high-level resistance to triclosan in *S.* Typhimurium that also increased the AMR and efflux pump activity, especially to enrofloxacin and sulphamethoxazole-trimethoprim (Gantzhorn et al., 2015). Genes encoding ESBLs and/or plasmid-mediated quinolone resistance genes and quaternary ammonium compound not only co-localize resistances, but can also co-transferred in *E. coli* (Li et al., 2017b).

Natural DNA transformation is an another mechanism that facilitates the transfer of genetic elements carrying AMR determinants (Winter et al., 2021). This mechanism involves the uptake of free DNA from the environment by the bacterial cells. Homologous recombination enables the internalization and integration of exogenous DNA into the host chromosome. The uptake of DNA is mediated by several proteins that help in DNA-binding, fragmentation, and transportation. For example, in *C. jejuni* Cj1211 strain, a *Helicobacter pylori* ComH3 homolog was found to be responsible for the acquisition of resistance to kanamycin or tetracycline (Jeon et al., 2008). The DNA uptake mechanism explains the acquisition of large *A. baumannii* resistance islands (AbaR4 and AbaR1) for carbapenem resistance in *A. baumannii* (Godeux et al., 2022). Intra-species genetic transformation from *Campylobacter coli* to *C. jejuni* was identified through the transfer of *ermB* gene that encodes resistance to macrolides in the chromosomal MDR GI (Qin et al., 2014).

Some of the susceptible pathogens may acquire indirect resistance assisted by the other juxtaposed antibiotic-resistant bacterial population. In *in vitro* conditions, it was shown that the presence of AMR bacteria expressing antibiotic-modifying/degrading enzymes such as β-lactamase, erythromycin esterase, tetracycline destructases, chloramphenicol acetyltransferase or the other cytoplasmic antibiotic-degrading enzymes that catalyze energy-consuming reactions, benefitted the susceptible population (Nicoloff and Andersson, 2016).

## Coordinated regulation of AMR with other functional genes

The dynamics of AMR evolution depend on the interactions between various cellular components. Multifunctional proteins are essential for bacterial fitness, AMR, and virulence. The catalytic domain EptC functions as a phosphoethanolamine transferase in *C. jejuni*. Mutational analysis of EptC indicated its importance not only for flagellar assembly and motility, but also for conferring polymyxin-B resistance (Fage et al., 2014). However, the function of EptC cannot be generalized, as it may differ among bacterial species.

Progression of AMR includes alterations in many cellular functions, such as membrane transport, transcription, translation, metabolic activity, etc. These important changes are achieved through a well-coordinated manner to accomplish greater fitness in bacteria. For e.g., β-lactam and aminoglycoside susceptibility in *E. coli* is enhanced by the changes in the proton- motive force through the cell membrane and respiratory activity (Lázár et al., 2013; Suzuki et al., 2014). Metabolic homeostasis plays an important role in AMR. Alteration of metabolic activity causes *S. aureus* to develop resistance to daptomycin and also presents innate immunity. This change was typified by a modification in the anionic membrane phospholipid structure, prompted by point mutations in the *cls2* that encodes a cardiolipin synthase (Jiang et al., 2019). The mutation in the *cls2* helps *S. aureus* to escape neutrophil chemotaxis, which is facilitated by the decrease in bacterial membrane phosphatidylglycerol.

Triosephosphate isomerase (TpiA) acts an important role in the carbon metabolic network, pathogenesis, and aminoglycoside resistance in *P. aeruginosa* (Xia et al., 2020). TpiA converts glyceraldehyde 3-phosphate to dihydroxyacetone phosphate, which is an important step in the bacterial glucose metabolism. Mutation in *tpiA* increases the carbon metabolism, but decreases the expression of the type III secretion system (T3SS), and reduces the bacterial resistance to aminoglycosides. A mutation in another carbon metabolism encoding gene in *P. aeruginosa*, *crcZ* with a *tpiA* mutant background restores the expression of T3SS and conferred aminoglycoside resistance (Xia et al., 2020). In *E.coli*, PAP2 protein (phosphatidic acid phosphatase) is involved in the phosphatidic acid pathway, which is important in the function of MCR-1 (Choi et al., 2020).

In *E. faecalis*, genes encoding the phospho-N-acetylmuramoyl-pentapeptide-transferase (*mraY*), penicillin-binding protein (*pbpC*), biosynthesis of peptidoglycan layers UDP-N- acetylmuramoylalanyl-D-glutamate 2,6-diaminopimelate ligase (*murEGD*) shared more direct interactions in the network analysis (Naha et al., 2020). The function of these genes is interrelated to the resistance mechanisms for β-lactams, ABC transporter MDR efflux pumps, active modifications to cell wall organization, and peptidoglycan pathways. Sulfhydryl reagent variable (SHV) β-lactamases contain a large number of allelic variants that include both ESBL and non-ESBL. SHV-11 and SHV-12 are commonly found in many members of the Enterobacteriaceae, especially in *K. pneumoniae* expressing extensive drug-resistance. Gene network analysis using *bla*_SHV-11_ showed that the MDR is associated with *gyrA*, *parC*, *glsA*, *osmE*, *yjhA*, *yhdT*, *rimL*, *pepB*, and KPN_00437, and KPN_01875 ORFs that are responsible for several DNA metabolic processes, DNA repair, and stress response (Miryala et al., 2020). Of these, *gyrA*, *parC*, *gyrB*, *parE*, *recA*, *dnaA*, *polB*, *dnaK*, *mutS*, and *dnaN* formed center nodes in the network that comprise more than 40% of the total interactions. Among the AMR associated genes such as *gyrA*, *nfxB*, *rnfC*, *parC*, and *parE*, only the *gyrA* mutation was predominantly important (Miryala et al., 2020). Additional mutations in any one or more of these genes further increased the ciprofloxacin MIC in a *gyrA* mutant background (Rehman et al., 2021a). Thus, an epistatic network of gene interactions can play a significant role in fluoroquinolone resistance.

AmpR regulates the expression of several genes that are involved in different pathways, including β-lactam and non-β-lactam resistance, virulence, quorum sensing, protein phosphorylation, and other physiological processes. Loss of *ampR* function in *P. aeruginosa* enhances its virulence, thereby confirming the existence of a co-regulatory network of β- lactam resistance, overexpression of the exopolysaccharide (alginate production), and quorum sensing (Balasubramanian et al., 2011).

Antimicrobial stress can induce genetic modifications in functional proteins related to bacterial metabolism. Gentamicin-induced resistance in *V. alginolyticus* has caused mutations in the metabolism-associated genes: *ratA, envZ, cydA1*, *malF* (encoding cyclase, osmolarity sensor protein, cytochrome d-terminal oxidase subunit-1, maltose transporter, respectively) and the *aceF*, encoding a subunit of pyruvate dehydrogenase, which is a two protein complex (Zhang et al., 2019). In addition to the reduced gentamicin uptake, these mutations decreased the pyruvate cycle, redox state, and membrane potential through the Na^+^ translocating the NADH-quinone oxidoreductase system (Zhang et al., 2019).

In *E. coli*, *arcA* (ArcA/B two-component system and a global regulator of gene expression under microaerobic/anaerobic conditions) and *gutM* (DNA-binding transcription factor) deletions showed inhibition of resistance to cefixime, ciprofloxacin, and chloramphenicol. Deletion in *arcA* activates aerobic respiration and the production of ROS that support antibiotic-mediated cell killing (Horinouchi et al., 2020). *gutM* regulates genes related to glucitol utilization are involved in the biofilm formation.

The base excision repair system plays an important role in preventing mutations that influence oxidative DNA damage. A base excision repair homolog encoded by the gene *cj0595c* in *C. jejuni* has shown an endonuclease III activity for maintaining genomic stability (Dai et al., 2019). Inactivation of *cj0595c* resulted in higher rates of spontaneous fluoroquinolone-resistant and oxidative stress-resistant mutants in the *gyrA* and *perR* genes, respectively (Dai et al., 2019). The four-gene operon *msaABCR* regulates vancomycin resistance, biofilm formation, and virulence in *S. aureus*. MsaB, a protein product of this operon, transcriptionally regulates the expression of the *crtOPQMN* operon and the *ohr* gene (encodes organic hydroperoxide resistance), which prevents oxidative stresses (Pandey et al., 2019). Deletion of *msaABCR* decreases vancomycin MIC in *S. aureus* and downregulates several genes involved in resistance against oxidative stress generated by organic hydroperoxides (Samanta and Elasri, 2014). Suprainhibitory concentration of ciprofloxacin in *Campylobacter* induced significant changes in the gene expression of *mfd*, which encodes a transcription-repair coupling factor involved in strand-specific DNA repair (Han et al., 2008). A mutation in the *mfd* caused a several-fold reduction in the rate of spontaneous mutation to ciprofloxacin resistance, while overexpression of this gene increased the mutation frequency. *V. cholerae* exposed to subinhibitory concentrations of ciprofloxacin, tetracycline, and azithromycin induces the SOS response. This response increases the expression of *recA* and *lexA* genes as well as increased the frequency of transfer of SXT elements to other bacterial population (Mohanraj and Mandal, 2022).

## The two-component system

The two-component system (TCS) is a signal transduction regulatory circuit in bacteria, which acts as an autoregulatory mechanism with a membrane-bound histidine kinase for sensing environmental signals and a cytoplasmic response regulator. The activated response regulator functions as a transcription factor that facilitates the cellular response/cell physiology through differential expression of target genes. This regulatory action activates antimicrobial defense and changes the cell physiology to increase the resistance. TCS modifies cell surface and permeability, enhances biofilm formation, and upregulates antimicrobial-degrading enzymes. Some important TCS includes VanSR-vancomycin^R^ in *S. aureus*, and *E. faecalis*; PmrAB and PhoPQ-polymyxin-B^R^/colistin^R^ in most of the Gram-negative pathogens; AmgRS- aminoglycosides^R^, CopRS-β-lactam/carbapenem^R^ in *P. aeruginosa;* BaeSR-ceftriaxone^R^ in *E. coli* and *Salmonella*; CesRK-β-lactam^R^ in *L. monocytogenes*, CreBC- β-lactam^R^, BlrAB- β- lactam^R^ in *Aeromonas* spp., etc. (Bhagirath et al., 2019; Tierney and Rather, 2019; Huang et al., 2020).

The TCS PhoPQ and PmrAB in *K. pneumoniae* and *E. coli* are controlled by additional proteins, MgrB, a negative regulator of PhoQ, and PmrD-a connector protein that activates PmrAB in response to PhoPQ stimulation (Chen et al., 2021). In regulating the polymyxin resistance, PmrD is dependent on the network organization as well as PmrB stimulation. The TCS regulator CpxAR has been reported to contribute to the MDR in several bacteria. In *S*. Typhimurium, CpxR regulate the colistin susceptibility *via* PmrAB and PhoPQ regulatory systems (Zhai et al., 2018). Generally, the TCSs PhoP-PhoQ and PmrA-PmrB control modification of lipid A of lipopolysaccharide (LPS) for adaptive peptide resistance under low Mg^2+^ conditions. In *P. aeruginosa*, CzcRS increases the resistance to heavy metals as well as carbapenems (Marguerettaz et al., 2014). ParRS directs increased resistance to aminoglycosides and the other polycationic antibiotics. This TCS also activates the LPS modification operon *arnBCADTEF* in the presence of subinhibitory concentrations of polymyxin and colistin (Fernández et al., 2010).

The cephalosporin sensitivity response regulator (CesR) and the cephalosporin sensitivity histidine protein kinase (CesK), encoded in the gene downstream of *cesR* influences the fitness of *L. monocytogenes* against cell wall-affecting β-lactam antimicrobials (Kallipolitis et al., 2003). In-frame deletions of *cesR* or its downstream positioned *cesK*, increases the sensitivity to β-lactams. The two-component transduction system of *L. monocytogenes* was found to be associated with tolerance to the cephalosporin family of antimicrobials and also to the lantibiotic nisin, which is a natural preservative, used in many food products (Cotter et al., 2002). TCS also enhances the growth of some of the pathogens in the presence of antimicrobials. Ferritin-like protein facilitates β-lactam tolerance and intrinsic resistance to cephalosporins in *L. monocytogenes*. However, the phosphate-response regulator (*phoP*) and an AraC/XylS family transcriptional regulator (*axyR*) that help the growth of *L. monocytogenes* in the presence of β-lactams have no role in the susceptibility or the tolerance to these antimicrobials (Krawczyk-Balska et al., 2012). CroSR is required for cephalosporin resistance in *E. faecalis*. To support this TCS, histidine-phosphorylatable phosphor carrier protein was identified to control the signaling system that modulates cephalosporin resistance (Snyder et al., 2014). CroSR-HPr association integrates antibiotic resistance and the bacterial nutritional status *via* various environmental stimuli.

## Clustered regularly interspaced short palindromic repeats system

Clustered regularly interspaced short palindromic repeats (CRISPR) coupled with CRISPR- associated proteins (Cas) protect bacteria against invading DNA (transposons, bacteriophages, and plasmids) in a programmed, sequence-specific manner. In the absence of the CRISPR/Cas system, many bacterial pathogens carry several ARGs. Recent findings suggest that this system can be useful in controlling the progression of AMR in bacteria and can be repurposed to control the antimicrobial resistance by the expression of self-targeted CRISPR that redirects endogenic CRISPR-Cas3 activity. However, several factors could contribute to resistance against the antimicrobial action of CRISPR-Cas. This includes spontaneous mutations in the Cas genes or the other target sequences, spacer deletions due to the homologous recombination between the repeats, expression of the anti-CRISPR (*acr*) gene in the host genomes, and repressed expression/activity of *cas*-encoded proteins (Gholizadeh et al., 2020). In addition, cell to cell communications within a bacterial population through disruption of two major quorum-sensing genes, *las* and *rhl* has been shown to reduce CRISPR-Cas activity (Høyland-Kroghsbo et al., 2017).

The role of CRISPR-Cas system in AMR is not fully investigated (Shabbir et al., 2018). Recent reports indicate the importance of this system in AMR in many pathogens. Transcriptome analysis of Δ*cas9* mutant *C. jejuni* was found to be more sensitive to antibiotics than its wild strain (Shabbir et al., 2018). Sequence-based analysis indicates the association between CRISPR-Cas and ARGs in *A. baumannii, E. faecium, P. aeruginosa*, and *S. aureus* (Mortensen et al., 2021). Homologous CRISPR spacers found in *S.* Enteritidis strains might be associated with MDR (Haider et al., 2022). Moreover, CRISPR spacers control several genes that participate in the maintenance of the membrane integrity to overcome different stresses including AMR.

## Virulence and AMR

Bacterial virulence factors are closely associated with various molecules produced by the pathogens, mainly to escape their host defense, survival and proliferation before colonization. Existence of a strong correlation between ARGs and the expression of many virulence factors have been reported in *S. aureus* (Obaidat et al., 2015), *E. coli*, and *K. pneumoniae* (Yang et al., 2015). Pan-resistant and MDR strains of many pathogens generally show enhanced virulence *in vivo* and *in vitro* (Qin et al., 2020). Apart from gene-encoded virulence, different phenotypic traits and cellular assemblies are also considered as virulence factors. Bacterial exo/endotoxins, specific enzymes, exopolysaccharides, flagella, fimbriae/pilli, capsules, LPS, glyco/lipoproteins are important in the pathogenesis. Intracellular changes in metabolic networks directed by protein regulators/receptors and non-coding regulatory RNAs are considered as virulence derived factors. It is important to address gene consortium, as many ARGs can act in tandem with virulence genes and other MGEs. From several investigations, it is evident that AMR and pathogenesis often exist concurrently. As shown in **Figure 4**, several regulatory network mechanisms exist involving AMR and virulence. However, their direct genomic association has not been well established due to complex network among the diverse species of bacterial pathogens.

In the *in vivo* colonization model, fluoroquinolone-resistant strain of *C. jejuni* with the C257T mutation in the *gyrA* outcompeted its clonally related fluoroquinolone-susceptible counterpart in the absence of any antimicrobial pressure (Luo et al., 2005). This enhanced fitness of the *C. jejuni* mutant strain also modulated its *in vivo* fitness. Co-existence of certain species of pathogens might support each other for enhanced growth, AMR, and virulence. The swine pathogens, *Streptococcus suis* and *Actinobacillus pleuropneumoniae* formed dual-species biofilm when co-cultured *in vitro*. Under this state, genes associated with exotoxins and adhesins were induced along with an increase in AMR (Wang et al., 2020b). These results suggest that the interspecies interactions may complement each other under specific conditions and play an important role in the AMR and disease progression. In the co-infection model, *P. aeruginosa* affects the *S. aureus* transcriptome and virulence with upregulation of several transporter encoding genes, especially, the Nor family of antibiotic pumps, leading to an increase in *S. aureus* AMR and an enhanced internalization within pulmonary epithelial cells (Briaud et al., 2019). During infection status, intrinsic and acquired ARGs provide a favorable *in vivo* fitness to several pathogens like *P. aeruginosa*, *A. baumannii*, and *V. cholerae* (Roux et al., 2015).

The outer membrane (OM) in Gram-negative bacteria acts as a barrier to different classes of antimicrobials. Colicin receptor CirA is an OM catecholate siderophore receptor involved in the iron uptake and colicin1A/B-mediated competitive killing in *S*. Enteritidis. The inactivation of the *cirA* gene caused impaired antibiotic resistance and also abruptly decreased the biofilm formation, adhesion, invasion of human epithelial cells, and proliferation in mouse macrophages (Zhang et al., 2020). Decreased expression of *oprD* that encodes the OM porin protein OprD increases carbapenem resistance and virulence in *P. aeruginosa* (Abdelraouf et al., 2011). In hypervirulent clinical *K. pneumoniae, r*educed expression of major OM porins (OmpK35/36) was found to be associated with carbapenem-resistant (Zhang et al., 2015b).

An assemblage of negative regulator MgrB, which is a transmembrane protein initiate the PhoPQ signaling system. Alteration of *mgrB* not only confers colistin resistance in *K. pneumoniae*, but also induces PhoPQ-governed lipid-A remodeling that enhances its virulence by reducing antimicrobial peptide susceptibility as well as host defense response (Kidd et al., 2017). The chaperone Sur (survival) is involved in the biogenesis of many OMPs and is an important member of the periplasmic network due to its genetic association with the β-barrel assembly machinery complex. The deletion mutant of *sur* in MDR *P. aeruginosa* increased susceptibility to ampicillin/sulbactam, ceftazidime, fosfomycin, and vancomycin and decreased serum resistance that led to a reduction of virulence *in vivo* due to several changes in the OMPs (Klein et al., 2019). LPS and capsular polysaccharide (CPS) plays a crucial role in the pathogenicity of bacteria. *gnaA* encodes UDP-*N*-acetylglucosamine C-6 dehydrogenase is confined in the CPS in *A. baumannii.* Higher expression of this gene plays an important role in virulence and resistance to carbapenems and cephalosporins (Xu et al., 2019). In *C. jejuni*, the truncation of LPS greatly reduced the intrinsic resistance to erythromycin due to enhanced permeability (Jeon et al., 2009).

Chemotaxis and swimming motility are associated with virulence in many pathogens that allow them to reach particular sites within the host (Josenhans and Suerbaum, 2002). Deletion of kanamycin resistance *aphA1*-encoding aminoglycoside-phosphotransferase limits the swimming ability of *S.* Typhimurium. Complementation with the respective allele of *aphA* restored the swarming phenotype, indicating the additional role of *aphA* in this pathogen (Brunelle et al., 2017). In many Gram-negative opportunistic pathogens, there is a strong interplay between β-lactamase regulated peptidoglycan and fitness/virulence. Peptidoglycan-remodeling enzymes are essential for the precise assembly of the T3SS machinery, flagella, invasiveness, and activation of the innate immune response (Juan et al., 2017).

Subinhibitory concentrations of tetracycline in *S.* Typhimurium increased its colonization ability and expression of epithelial cell invasion by up-regulating the *hilD* and *hilA* genes. These two genes regulate SPI-1 and also the *fliC*, *fliD*, *fur*, *motA* and *motB* genes that are associated with motility, regulation of iron acquisition systems, acid tolerance (Weir et al., 2008). Exposure of *Enterococcus faecium* to subinhibitory concentrations of ciprofloxacin upregulates the *qnr* gene that encodes quinolone resistance and collagen adhesin gene (*acm*). This mechanism favored not only the fluoroquinolone resistance, but also the adhesion ability of *E. faecium* (Sinel et al., 2017). In Shiga toxin-producing *E. coli* treated with subinhibitory concentrations of ciprofloxacin and tigecycline, a 6-8-fold increase in the expression levels of *stx1* and *stx2* has been reported (Rehman et al., 2021b).

Hemolysin is one of the major virulence factors in *S. aureus*. A significant relationship exists between the presence of α-hemolysin encoding gene (*hla*) and the antibiotic resistance of *S. aureus* isolates, especially to erythromycin and penicillin (Motamedi et al., 2018). The AMR activity was not noticed in deletion mutants of *hla*. The *oqxAB* gene that encodes the multidrug efflux pump OqxAB, is located on the chromosome and/or in plasmids flanked by IS*26*-like elements in several members of Enterobacteriaceae. This gene can co-spread both antimicrobial resistance genes (*bla*_CTX-M_, *rmtB* and *aac(6’)-Ib*) and virulence genes (Li et al., 2019). *S. suis* cause septicemia and meningitis in swine and humans. In MDR *S. suis*, a potential efflux pump SstFEG function from the upstream of the bacitracin-resistance genes *bceAB* and *bceRS.* The network of BceAB, BceRS, and SstFEG not only regulates the bacitracin efflux optimally, but also involved in enhanced colonization and higher virulence of *S. suis* (Ma et al., 2019b).

ESBL-plasmid-carrying curli fimbriae support adhesion and are associated with the internalization of epithelial cells. The plasmids of ESBL-producing *E. coli* from pandemic lineages have the potential to influence chromosomal gene expression; particularly the curli fimbriae-specific gene *csgD* contributes to their virulence and pandemic success (Schaufler et al., 2016). In clinical strains of *K. pneumoniae*, the existence of *mrkD* (type 3 fimbrial adhesion) was found to be associated with the *bla*_DHA-1_ (Soltani et al., 2020).

AmpC ß-lactamase regulator AmpR regulates several virulence determinants in *P. aeruginosa*, including pyocyanin-a redox-active compound and elastases that cause severe tissue damage during infection (Balasubramanian et al., 2012). Cysteine phosphorylation of staphylococcal accessory regulator A (SarA/MarR family) mediates vancomycin resistance and virulence in the mouse model (Sun et al., 2012). Hypervirulent MDR *K. pneumoniae* from diseased pig carried an ICE*Kp*SL1, which had an intact *Yersinia* high-pathogenicity island for the expression of a high-virulence phenotype involving biosynthesis, regulation and the transport of the siderophore, yersiniabactin (Liu et al., 2019). This ICE could be excised from the host chromosome in the form of a circular intermediate before it spread to other bacteria. In *K. pneumoniae, bla*_CMY-2_ was significantly correlated with the presence of *iutA* (aerobactin siderophore ferric receptor) and *rmpA* (positive regulator of extracapsular polysaccharide synthesis that confers a mucoid phenotype) virulence genes (Soltani et al., 2020).

Biofilm formation is one of the virulence mechanisms that has a link with AMR. This association has been reported for gentamicin and ceftazidime resistance in *E. coli*, piperacillin/tazobactam, and colistin resistance in *K. pneumoniae*, and ciprofloxacin resistance in *P. aeruginosa* (Cepas et al., 2019). The BfmRS two-component system is involved in the regulation of biofilm production and is a global regulatory system that mediates the virulence-antibiotic resistance network. BfmRS-protects *A. baumannii* from serum complement killing through global transcriptional modulation of envelope biogenesis and defense pathways. This mechanism causes severe systemic disease in mice, and increased resistance to several antimicrobials, including the β-lactams (Geisinger et al., 2018). ESBL resistant *K. pneumoniae* strains expressed increased biofilm formation and resistance to serum killing through the *iss* gene (increased serum survival) as observed from mice mortality (Zhang et al., 2015b; Gharrah et al., 2017).

## Conclusions and perspectives

Selection pressure conferred by the use of antibiotics in human medicine, animal farming, the food industry, and agriculture practice leads to a significant increase in AMR and a constant accumulation of ARGs in bacteria. Effective survival in the environment and colonization in many hosts are important components that help the pathogens to survive and spread the AMR. Controlling the spread of AMR is of prime concern that must be addressed to ensure that the treatment methods against infectious diseases remain effective. Numerous ARGs that confer resistance have been identified and characterized. The ARGs are highly intertwined and robustly regulated in a specific or through multiple bacterial genome/proteome networks. A deeper understanding of such complex networks can help in uncovering the resistance pathways and identification of specific aspects of intervention through resensitization of MDR pathogens. The advancements made in whole genome sequencing and the application of bioinformatics tools have identified several ARGs and thrown light on their role in bacterial environmental fitness, ecology of resistance, and pathogenesis. Some of this information available in public domains can be effectively used in future functional studies. Through the genomic/proteomic network, several components that play a role in resistance, as well as its controlling factors can be identified in the context of preventing the AMR and HGT.

## Author contributions

TR, AG and AKM conceived and structured the review. TR and AG wrote the manuscript. GC, AKM, SD, and S-IM critically analyzed the presentation. All authors discussed the results, and reviewed and commented on the manuscript. All authors contributed to the article and approved the submitted version.

## Funding

This work was supported in part by the Indian National Science Academy (INSA), Japan Initiative for Global Research Network on Infectious Diseases (J-GRID), the Ministry of Education, Culture, Sports, Science and Technology in Japan, the Japan Agency for Medical Research and Development (AMED; Grant No. JP22wm0125044), Indian Council of Medical Research, and the National Academy of Sciences (NASI), India. TR is INSA-Senior Scientist, AG is J. C. Bose Chair Professor of the NASI, India.

## Acknowledgments

We thank R. Dharanidharan, Medical Biotechnology and Immunotherapy Unit, Faculty of Health Sciences, University of Cape Town, South Africa for the preparation of figures using BioRender.com.

## Conflict of interest

The authors declare that the research was conducted in the absence of any commercial or financial relationships that could be construed as a potential conflict of interest.

## Publisher’s note

All claims expressed in this article are solely those of the authors and do not necessarily represent those of their affiliated organizations, or those of the publisher, the editors and the reviewers. Any product that may be evaluated in this article, or claim that may be made by its manufacturer, is not guaranteed or endorsed by the publisher.
